# EMT or EMT-Promoting Transcription Factors, Where to Focus the Light?

**DOI:** 10.3389/fonc.2014.00353

**Published:** 2014-12-16

**Authors:** Stéphane Ansieau, Guillaume Collin, Louise Hill

**Affiliations:** ^1^INSERM UMR-S1052, Centre de Recherche en Cancérologie de Lyon, Lyon, France; ^2^CNRS UMR5286, Centre de Recherche en Cancérologie de Lyon, Lyon, France; ^3^LabEX DEVweCAN, Lyon, France; ^4^UNIV UMR1052, Lyon, France; ^5^Centre Léon Bérard, Lyon, France

**Keywords:** EMT, escape from fail-safe programs, EMT-TFs, plasticity, EMT-promoting

The epithelial-to-mesenchymal transition (EMT) is commonly considered as a main driving force of the metastatic cascade. An impressive series of experiments and observations worldwide supports its pivotal role in promoting cancer cell dissemination at the invasive fronts of tumors, intravasation, cell survival in fluids, and extravasation, with secondary site colonization being the only step requiring a return to an epithelial phenotype (1). Beyond this, commitment of epithelial cells into an EMT program has been associated with resistance to chemo-, radio-, and hormono-therapies, definitively extending the interest of studying EMT from the embryologist community to the oncology and medical fields. Obviously, EMT turns out to constitute a transdifferentiation program, allowing epithelial cells to escape from numerous stresses, including mechanical constrains, hypoxia, nutrient depletion, and unfortunately therapeutic treatments (1). EMT is orchestrated by a restricted number of transcription factors mainly the three Snail, Twist, and Zeb families (EMT-TFs). With inclusion of miRNAs, these factors constitute a complex interactome, able to sensor multiple signals received from the proximal microenvironment and relay them onto gene expression (2, 3).

Generally undetectable in adult epithelial cells in homeostatic conditions, EMT-TFs were found to be recurrently expressed in various types of cancers including multiple carcinomas, an expression often associated with a high metastatic risk. An outstanding amount of work has been performed over the last years evaluating their relative contribution to the initiation and maintenance of the EMT process, with a significant impact on the prognostic value occurring following to their detection in primary tumors or in disseminated cancer cells (4, 5). Does this mean that the oncogenic potential of these factors in epithelial cells only rely on their ability to promote EMT or does their oncogenic activity extend further than EMT induction?

By performing *in vitro* functional assays, we and others firstly demonstrated that several of these embryonic transcription factors, including the TWIST proteins, alleviated fail-safe program induction. Through this, they were shown to cooperate with mitogenic oncoproteins such as RAS and N-MYC in promoting cell transformation *in vitro*, as well as in lung and breast carcinoma development in transgenic mouse models (4, 6–9). The underlying mechanisms have been largely explored. The TWIST1 protein was found to directly interact with p53 and to destabilize the oncosuppressive protein by altering specific post-translational modifications (10). Furthermore, TWIST1 alleviates induction of cyclin-dependent kinase inhibitors (*CDKN1A*, *CDKN2A*, and *CDKN2B*), thereby sustaining cell proliferation (8, 11). These pleiotropic properties probably provide TWIST proteins unique properties. As TWIST proteins experimentally are inefficient in triggering EMT as compared with SNAIL and ZEB proteins, their main functions may consist in protecting cells from fail-safe programs during the EMT-associated genetic reprograming. In this respect, they might be considered as survival factors rather than EMT inducers. Although SNAIL and ZEB proteins, unlike the TWIST transcription factors, fail to prevent HRAS^G12V^-induced senescence in murine embryonic fibroblasts (B. Gras, unpublished data), we obviously cannot exclude that they facilitate the escape from oncogene-induced fail-safe programs in other cellular settings and/or experimental conditions. In support of this assumption, ZEB proteins were indeed reported to protect lung cancer cells from EGFR-induced senescence through their ability to down-modulate *CDKN1A* and *CDKN2B* (12). In line with this observation, ZEB1 was demonstrated to be positioned downstream of RB and to contribute to fibroblast immortalization induced by RB and RB-like protein depletion (13). Enforced expression of SNAIL or ZEB proteins in non-transformed mammary epithelial cells, and the consequent activation of RAS-downstream pathways, predominantly triggers EMT. Nonetheless, it also accidentally promotes the commitment of cells into a senescence program, unveiled by their SA-β-galactosidase activity (B Gras and SA, unpublished data). This observation is consistent with the reported anti-proliferative properties of SNAIL and ZEB proteins in epithelial cells (14) and with the recognized antagonism between cell proliferation and dissemination. It additionally gives a rationale to the restricted staining of these transcription factors to the tumor-stromal interface, stabilized by microenvironmental EMT-permissive conditions (15–17). The need to maintain ZEB and SNAIL proteins at a basal level to sustain epithelial cell proliferation is difficult to reconcile with a role in fail-safe program escape. Nonetheless, the fact that the EMT-promoting and fail-safe program inhibition induced by ZEB1 requires different levels of protein expression (18) suggests that a low protein level (and likely not always detectable by immunohistochemistry) is not incompatible with such a function. Most of these transcription factors are particularly unstable, subjected to post-translational modifications and thereby transiently stabilized and activated. Knockdown experiments, rather than stable enforced expression, are thus warranted to gain further insight into their functions. Such an approach has successfully been employed to emphasize the temporally distinct functions of SNAIL1 and TWIST1 during the TGFβ-driven EMT (19). Interestingly, human sarcomas were recently shown to display high SNAIL1 expression and SNAIL1 was demonstrated to control the tumorigenic properties of mesenchymal cells (20). In this tumor progression model, the anti-apoptotic properties of SNAIL proteins may provide cells a survival advantage, which would enhance their potential to undergo neoplastic transformation. Additionally, the SNAIL1 protein has been reported to alleviate the differentiation of multipotent mesenchymal stem cells (21), the cells of origin of certain sarcomas [reviewed in Ref. (22)].

By facilitating the escape from fail-safe programs, TWIST proteins may not only contribute to facilitate tumor initiation but also provide cancer cells with proliferation and survival advantages. Obviously, numerous cancer cell lines from various tumor types including breast and lung carcinoma, sarcoma, and neuroblastoma were found to remain dependent on TWIST1 for their survival (7, 8, 11). As already mentioned, ZEB1 was similarly shown to abrogate latent EGFR-induced senescence in lung carcinoma cells (12). The addiction to a specific embryonic transcription factor may be determined by the nature of the original insult, e.g., in murine pancreatic epithelial cells, TWIST1 is induced in response to K-RAS activation and, avoids replicative senescence by turning-down *Cdkn2A* (23).

As an interconnected transcriptional network, expression of SNAIL, TWIST, and ZEB proteins induces a profound genetic reprograming of cells, with the corresponding consequences upon epithelial integrity undoubtedly constituting only a single facet of this remodeling. A brief overview of the induced genetic changes unambiguously highlights profound metabolic modifications and in support of this observation, SNAIL1 was shown to favor glycolysis, glucose uptake, maintenance of ATP production in hypoxic conditions and to reduce ROS production (24, 25). An additional consequence of this genetic reprograming is to afford cells a “plastic” configuration, with an exacerbated adaptability to hostile environments and an ability to quickly respond to their needs. As an example, enforced expression of TWIST1 in mammary epithelial cells poorly impacts on cell morphology but significantly accelerates their commitment to EMT when submitted to TGFβ, an EMT-promoting cytokine (9). Cell plasticity similarly determines the ability of EMT-committed cells to return to an epithelial phenotype in a restrictive microenvironment, promoting their capability to colonize secondary sites (26, 27). In this regard, neither epithelial nor mesenchymal cells, the two end points of the process, are likely to constitute the most aggressive cells, with the partially reprogramed and semi-committed cells being the most likely to switch between an invasive and proliferative status.

Partial reprograming driven by the embryonic transcription factors likely places cells at the intersection of different destinies, their outcomes being likely dictated by intrinsic properties, and/or genetic events. When combined with key regulators of cell determination, such as the SOX9 transcription factors, cells further commit to a dedifferentiation process (28). Dedifferentiation also takes place, at least to some extent, when the embryonic transcription factors are combined with mitogenic activations, leading to the reacquisition of some stem-cell-like properties, including a self-renewal potential (9, 29). In support of this assumption, combined expression of TWIST1 and an activated version of RAS in murine luminal committed mammary epithelial cells invariably leads to the development of carcinomas of a particular subtype referred as “claudin-low” (9): a group of tumors with enriched EMT and stem-cell features and originally believed to arise from mammary stem cells (30). The link between embryonic transcription factors and stemness has been further exemplified by the detection of ZEB1 specifically in poorly differentiated pancreatic carcinomas and the demonstration of its role in maintaining stemness through repression of stemness-inhibiting miRNAs (31). Combined EMT and stemness induction at the invasive fronts of tumors has been proposed as a first rationale to explain the dissemination of single cancer stem cells, able to colonize distant sites and yield secondary tumors with full heterogeneity (32).

Strikingly, partial commitment into EMT (and presumably the transition to this plastic state) was also demonstrated as sufficient to accelerate epithelial cell transformation. Presumably, the genetic reprograming impacts on multiple mitogenic (e.g., activation of the RAS pathway) and oncosuppressive (e.g., down-modulation of the activity of the phosphatase PP2A) pathways (9, 33). Whether cell dedifferentiation contributes to the oncogenic properties of these embryonic transcription factors in non-epithelial cells remains poorly investigated, with the exception of melanocytes. These neural-crest derived cells endogenously express SNAIL2 and ZEB2, both of which activate *MITF* transcription and induce downstream target genes to promote cell survival and proliferation. Following activation of the NRAS/BRAF pathway, a driver mutation in melanomagenesis, a redistribution of the embryonic transcription factors takes place, with SNAIL2 and ZEB2 being replaced by *ZEB1* and *TWIST1*. These two embryonic transcription factors display opposite functions to SNAIL2 and ZEB2, by turning-down *MITF* expression and silencing the downstream differentiation program to rather favor cell migration (34, 35). Strikingly, modulation of the MITF rheostat is determinant for melanocyte transformation (36). The reversible redistribution of these embryonic transcription factors furthermore regulates the equilibrium between the proliferative and invasive states of melanoma cancer cells, and thereby dictates their ability to complete the metastatic process. In support of this expectation, ZEB2 was identified as essential for secondary site colonization (37). It is actually very likely that the ability to alleviate differentiation programs or to induce cell dedifferentiation will turn, in the near future, to be one of the main oncogenic functions of these embryonic transcription factors, with dedifferentiation being regularly associated with, and likely an integral part of, neoplastic transformation (29, 38, 39). In this regard, the recent demonstration of a pivotal role of SNAIL1 in sarcomagenesis and its functions in preventing mesenchymal stem cell differentiation (20) likely reflects this behavior.

Resistance to therapeutic treatments in carcinoma cancer cells has also recurrently been associated with EMT. While this resistance might result from multiple mechanisms, including metabolic changes impacting on pro-drug activation and drug exclusion through transporters, recent observations also suggest that embryonic transcription factors might directly be involved in the emergence of such resistant cells, independently of their EMT-promoting functions, through various mechanisms. In a recent study, Zhang and co-workers have demonstrated that the ZEB1 transcription factor triggers radioresistance in an EMT-independent manner. Stabilized through phosphorylation by ATM, ZEB1 interacts with USP7 and enhances its ability to deubiquitinilate and stabilize CHK1, thereby favoring recombination-dependent DNA repair (40). In line with this observation, ZEB2 was shown to prevent ATM/ATR activation in response to a genotoxic stress in an EMT-independent manner and constitutes a factor of poor prognosis in bladder cancer patients treated with radiotherapy (41). TWIST1 was also previously demonstrated to trigger chemoresistance in an EMT-independent manner through its ability to induce *AKT2* expression and to differently modulate the ratio between pro- and anti-apoptotic members of the BCL-2 family [reviewed in Ref. (42)]. Lastly, SNAIL1 and SNAIL2 proteins protect kidney epithelial cells and hematopoietic precursor cells, respectively, from radiation-induced apoptosis by interfering with p53-target gene activation (43, 44).

The relative contribution of EMT and EMT inducers to tumor development is like the chicken and the egg question. Nonetheless, these observations collectively highlight numerous specific EMT-independent functions of these transcription factors, which likely merit consideration in line with the EMT-driven program that promotes carcinogenesis (Figure 1). This non-exhaustive list of functions of the EMT inducers likewise reflects only the emerged part of the iceberg. As previously mentioned, the EMT-promoting and fail-safe program inhibition induced by ZEB1 requires different levels of protein expression (18). Furthermore, ZEB1 depletion in SNAIL1-expressing cells radio-sensitizes cells without affecting their commitment into an EMT process (40), likely unveiling a yet underestimated level of complexity. No doubt novel functions involving EMT-unrelated genetic programs induced in different cellular settings and protein expression levels will soon emerge as an additional oncogenic weapon of these factors. Their common denomination as EMT inducers will then be obsolete.

**Figure 1:**
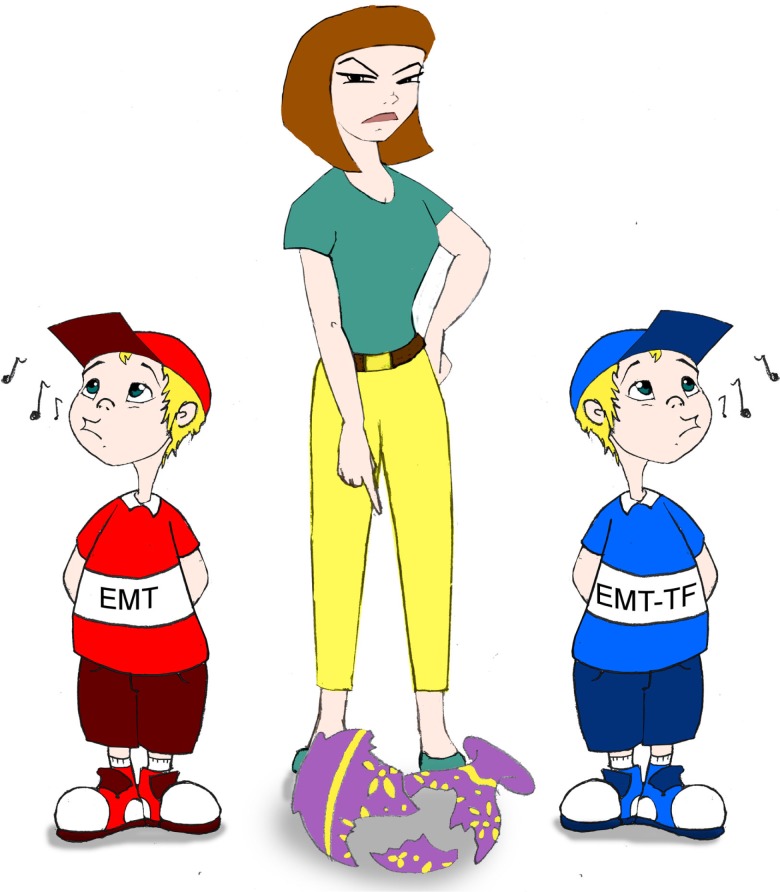
**As a mother trying to identify which of hers two sons broke the vase, scientists need to precisely determine the contribution of EMT and EMT-TFs in tumor development**.

## Conflict of Interest Statement

The authors declare that the research was conducted in the absence of any commercial or financial relationships that could be construed as a potential conflict of interest.
